# Effect of Duplication Techniques on the Fitting Accuracy of CAD-CAM Milled, 3D-Printed, and Injection-Molded Mandibular Complete Denture Bases

**DOI:** 10.3390/dj12020032

**Published:** 2024-02-02

**Authors:** Abdel-Naser M. Emam, Ahmed Ayman El-Esawy, Mohammed Hamad Alyami, Yasser Baraka, Mohammed M. Gad, Mohamed Ahmed Helal

**Affiliations:** 1 Prosthetic Dental Science Department, Faculty of Dentistry, Najran University, Najran 55461, Saudi Arabia; amemam@nu.edu.sa (A.-N.M.E.); mhalyame@nu.edu.sa (M.H.A.); 2 Department of Prosthodontics, Faculty of Dental Medicine, Sina University, El-Arish 45511, Egypt; dr.essawy92@gmail.com; 3 Department of Prosthodontics, Faculty of Dental Medicine, Deraya University, Al-Menia 61111, Egypt; yasser.baraka@deraya.edu.eg; 4 Department of Substitutive Dental Sciences, College of Dentistry, Imam Abdulrahman Bin Faisal University, P.O. Box 1982, Dammam 31441, Saudi Arabia; mmjad@iau.edu.sa; 5 Department of Prosthodontics, Faculty of Dental Medicine, Al-Azhar University, Cairo 11884, Egypt

**Keywords:** accuracy, complete dentures, denture base, duplication, CAD-CAM, milling, 3D printed

## Abstract

Background: Digital technology has been introduced in prosthodontics, and it has been widely used in denture duplication instead of a conventional denture duplication technique. However, research comparing different denture duplication techniques and how they affect the fitting accuracy of the denture base is scarce. Objectives: The aim was to assess the impact of duplication techniques on the accuracy of the fitting surface of computer-aided design and manufacturing (CAD-CAM) milled, 3D-printed, and injection-molded complete denture bases (CDBs). Methodology: This study involved fabricating a mandibular complete denture base with three marked dimples as reference marks (A, B, and C at the incisive papilla, right molar, and left molar areas) using a conventional compression molded technique. This denture was then scanned to generate a standard tessellation language (STL) file; after that, it was duplicated using three different techniques (milling, 3D printing, and injection molding) and five denture base resin materials—two milled CAD-CAM materials (AvaDent and IvoBase), two 3D-printed materials (NextDent and HARZ Labs), and one injection-molded material (iFlextm). Based on the denture base type, the study divided them into five groups (each with *n* = 10). An evaluation of duplication accuracy was conducted on the fitting surface of each complete denture base (CDB) using two assessment methods. The first method was a two-dimensional evaluation, which entailed linear measurements of the distances (A–B, A–C, and B–C) between reference points on both the scanned reference mandibular denture and the duplicated dentures. Additionally, a three-dimensional superimposition technique was employed, involving the overlay of the STL files of the dentures onto the reference denture’s STL file. The collected data underwent statistical analysis using a one-way analysis of variance and Tukey’s pairwise post hoc tests. Results: Both evaluation techniques showed significant differences in fitting surface accuracy between the tested CDBs (*p* ˂ 0.001), as indicated by one-way ANOVA. In addition, the milled CDBs (AvaDent and IvoBase) had significantly higher fitting surface accuracy than the other groups (*p* ˂ 0.001) and were followed by 3D-printed CDBs (NextDent and HARZ Labs), while the injection-molded (iFlextm) CDBs had the lowest accuracy (*p* ˂ 0.001). Conclusions: The duplication technique of complete dentures using a CAD-CAM milling system produced superior fitting surface accuracy compared to the 3D-printing and injection-molded techniques.

## 1. Introduction

A second denture meant to be a replica of the first is called a duplicate denture [1]. Many patients with complete dentures (CDs) ask their prosthodontist to make them duplicate dentures. These patients, especially when their own dentist cannot be reached, cannot bear the shame of being without a denture, even for a brief length of time, due to a denture fracture. Replacement dentures are generally similar to the patient’s existing ones [2]. For senior people, adapting to replacement dentures can be a constant challenge, especially when significant adjustments are needed to the CD’s occlusal and fitting surfaces [1,3]. Patients who have systemic diseases like Parkinson’s, dementia, and physical frailty are considered the most at-risk patients. The prosthodontist has to understand that the adaptability of elderly denture wearers is also influenced by their neuromuscular coordination, the health of the supporting tissues, and their desire to learn new skills [4,5]. 

The duplicate denture approach entails many methods designed to produce prostheses that resemble CDs [6]. There are several clinical and scientific techniques that, to varying degrees, can “copy” a prosthesis. The prosthodontist has to decide on one of the three denture surfaces (occlusal, polished, and fit) to reproduce or adjust depending on the clinical situation [7,8]. The traditional method often uses elastomeric impression or irreversible hydrocolloid materials to form a mold that acts as the denture’s opposite or negative representation. However, the traditional approach requires a lot of work and takes a long time [1].

Using PMMA’s pre-polymerized blocks, computer software, and five-axis milling, a new method for fabricating dentures using computer-aided design—computer-aided manufacturing (CAD-CAM) has been developed. With CAD-CAM technology, the digital impression and jaw relationship records were sent to dental labs for the commercial manufacturing of new CAD-CAM CDs [6].

Given the increasing use of dental scanners and 3D printers in dental offices, the replication technique is well positioned, precise, and more effective than conventional methods of treating older edentulous patients. The outcome is a high-quality prosthesis that has led to a reduction in the amount of time needed for adaptation and the frequency of adjustment appointments [9].

However, the processing of dentures with different fabrication methods could result in distortion, which can range from 0.45% to 0.9% linear distortion [6,10]. This deformation diminishes the ability of the denture base to adjust to the underlying soft tissue and leads to a reduction in retention, stability, and support. The patient’s comfort suffers as a result of this decrease in retention, stability, and support, and the clinician’s time spent in the chair increases due to the necessary changes [6]. At the time of denture production, the location and degree of dimensional change have been evaluated using a variety of techniques. These have included various complex two-dimensional and three-dimensional measures. Laser and contact scanners are now frequently used to measure the deformation of denture bases [6,11]. These methods enable the overlaying and analysis of scanned files utilizing cutting-edge software [6].

Digital denture duplication has been investigated in previous studies [12,13,14,15,16,17,18]. Clark et al. [12] reported that the digital replication technique of the exciting prosthesis is a proven technique with decreased laboratory steps and required chair time compared with conventional methods. Ammoun et al. [13] were among the first to use a dental technique in which the surface details of dentures were scanned, recorded, and saved for oral device fabrication and denture duplication, and they concluded that scanned files could be used for denture duplication as an alternative to the conventional technique. Renne et al. [14] reported many advantages of digital duplication such as a decreased number of visits, a subsequently decreased cost, decreased labor errors, increased dimensional accuracy due to dimensional changes in duplicating materials, and improved modifications and design of scanned files, in addition to high efficiency and suitable clinical solutions for many cases. Takeda et al. [15] reported that the digital replication technique takes less time and is more accurate compared to conventional duplication. 

Chen et al. [1] compared the accuracy of digitally duplicated dentures to conventionally duplicated ones and concluded that the digital technique showed high accuracy and effectiveness. Fekri et al. [16], compared, an ill-fitting maxillary denture duplicate with a well-fitted digitally duplicated denture and reported that digital duplication is a promising alternative to conventional duplication techniques. Alehaideb et al. [17], compared different desktops and intraoral scanners for digital denture duplication and concluded that all the scanners could duplicate dentures with high accuracy. In another study after creating a standard tessellation language (STL) file, dentures were duplicated using CAD-CAM milling and additive methods [12,13]. Although several studies [12,13,14,15,16,17] have assessed different techniques for digital denture duplication, different scanners, or provided a case report validating specific dental techniques, no studies have compared denture base materials and fabrication technologies in relation to digital denture duplications. 

Many studies have evaluated the influence of construction techniques on the properties of different denture base resins [11,18,19,20,21,22,23]. However, little data are available regarding the effect of different duplication techniques on the processing distortion of the CAD-CAM fabrication and injection molding techniques. Therefore, the primary goal of this in-vitro study was to assess the impact of duplication techniques on the accuracy of the fitting surface of CAD-CAM milled, injection-molded, and 3D-printed mandibular CDs. The null hypothesis was that the difference in the fitting surface accuracy of the CDs of the three different duplication techniques would be insignificant.

## 2. Materials and Methods

**Figure 1:**
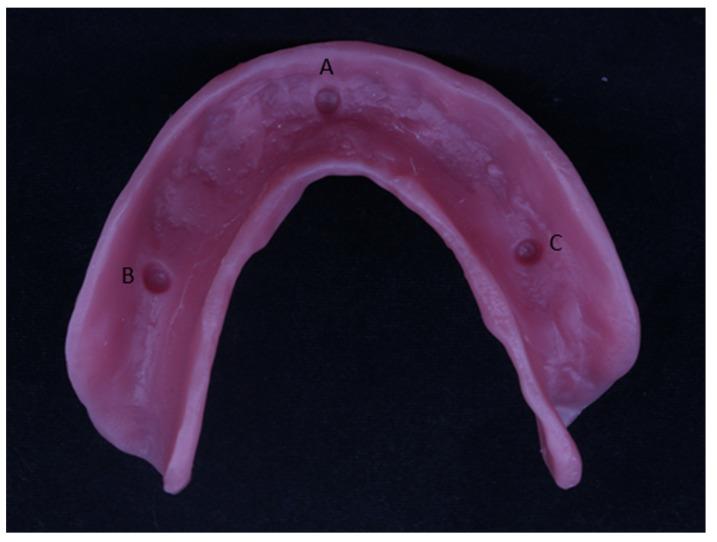
Reference denture model. A, B, and C are selected points on scanned mandibular denture bases for linear measurements.

**Figure 2:**
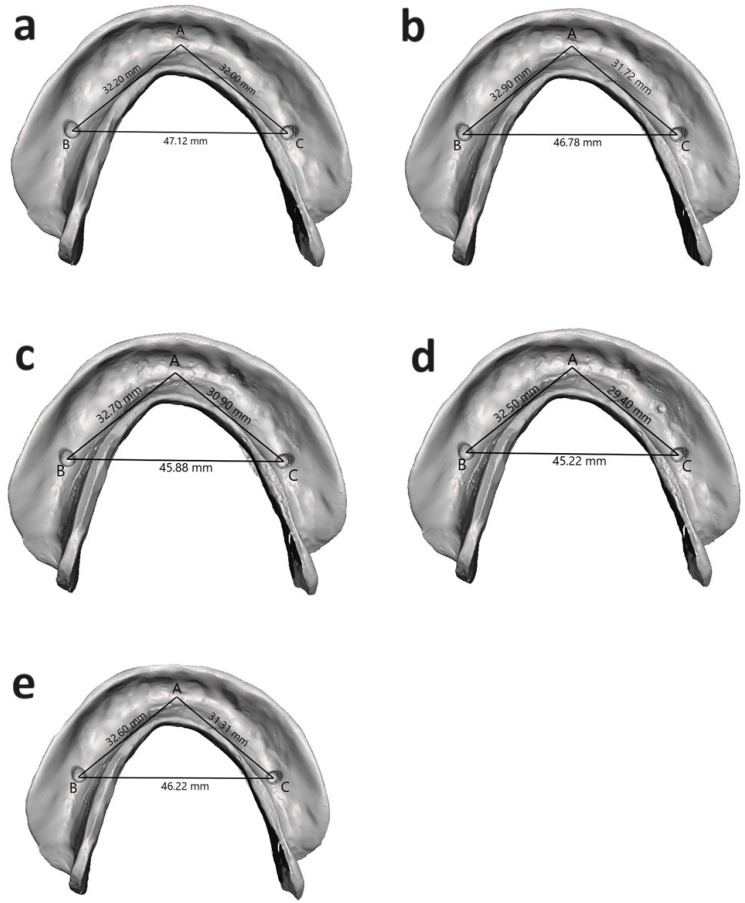
Representative images showing linear measurements between selected points on scanned mandibular denture bases. (**a**) AvaDent; (**b**) IvoBase; (**c**) NextDent; (**d**) Harz Lab; and (**e**) injected CD.

Based on the previous literature, the sample size was calculated [21] using G* power version 3.0.10, adopting an α level of 0.05, β level of 0.05 (i.e., power = 95%), and effect size of 0.759. The predicted sample size was 10 samples per group. A mandibular CD base constructed via the conventional compression molded technique was used as the reference CD base (Figure 1) to compare with the CD bases constructed using two different digital techniques and one injection molding technique [22]. Using no. 6 and no. 8 round burs, three dimples (2 mm × 2 mm) were created on the fitting surface of the reference CD base at three specific locations [24], which are represented by three reference marks: Point A, anteriorly at the midline (between 2 central incisors); Point B, on the right premolar areas; and Point C, on the site of the left premolar areas (Figure 2).

Fifty mandibular CDs were duplicated from a ready-made PMMA acrylic mandibular CD. These were categorized into five groups according to the denture base material, with 10 in each group: Group1 (G1), AvaDent; Group2 (G2), IvoBase; Group3 (G3), NextDent; Group4 (G4), Harzlab; and Group5 (G5), iFlext denture base materials.

### Denture Duplication

CAD-CAM milling copying technique: Twenty mandibular CD bases (CDBs) were constructed from AvaDent (G1, AvaDent, Global-Dental-Science-Europe, Tilburg, The Netherlands) and IvoBase (G2, IvoBase, Ivoclar, Vivadent-AG, Schaan, Liechtenstein) pre-polymerized denture base materials (*n* = 10). First, using the antiglare spray (Telescan-spray-white, DFS-Diamon-GmbH, Riedenburg, Germany) the prescribed mandibular CD was sprayed with a thickness of 7–10 microns. Then, the reference CD was scanned using a laboratory scanner (DOF, Swing, Seoul, Republic of Korea). The duplicated denture was then manufactured using CAD software (3 Shape A/S, 3 Shape Dental System, Copenhagen, Denmark). The definitive standard tessellation language (STL) design file was for duplicated CD fabrication. The STL file was then moved to the 5-axis milling machine (inLab MC X5, DENTSPLY, Sirona, PA, USA). A pre-polymerized PMMA block (98.5 mm in diameter × 30 mm in thickness) was milled with a minimum bur in a wet condition (Dentsply International Inc., York, PA, USA) with sizes of 0.5 mm, 1, and then 2.5 mm, while the connectors were adjusted to 10. After the duplicated dentures were milled with the aid of carbide burs, they were removed from the discs [10,20]. 

Three-dimensional (3D) printing duplicating technique: Twenty mandibular CDBs were constructed from NextDent (G3, NextDent Base, NextDent, Zetterberg, The Netherlands) and HARZ Labs (G4, HARZ Labs LLC Co., Ulitsa Tvardovskogo, Moscow) photopolymerized denture base materials (*n* = 10). The STL file was transferred to the 3D printing machine (ANYCUBIC photon, M3 plus, Shenzhen Technology, Co., Ltd, Quanzhou, Fujian, China). Ahead of the printing process, as per the manufacturers’ guidelines, the denture base resin underwent a 1 h mixing procedure using an LC-3D Mixer (NextDent, Soesterberg, The Netherlands). The denture bases were then vertically stacked (at 90-degree angles using approved denture base material and a digital light processor 3D printer at speeds ranging between 10 and 30 mm/h. The photopolymer substances were printed in a 3D pattern of ultra-thin layers (50 µm at 90° orientation) onto a building tray until the denture was fully formed. Each photopolymer layer underwent immediate curing by ultraviolet (UV) light. After the printing process, the denture bases were taken off the platform and cleaned for 5 min in a 99% isopropyl alcohol solution and an ultrasonic cleaner. Following this, they underwent a 30-minute post-curing process using a UV curing unit (LC-3D Print Box, NextDent, Soesterberg, The Netherlands) at 60 watts [10,20]. 

Injection-molding technique: Ten mandibular CDs were prepared using thermoplastic PMMA acrylic resin (G5, iFlextm, TCS Dental, Inc., Signal Hill, CA, USA) according to the manufacturer’s guidelines. A flask slightly larger than the reference denture was selected. Two wax sprues with 9.5 mm diameters were posteriorly attached to the border of the denture (one on each side). The lower half of the impression flask (TCS Dental, Inc., Signal Hill, CA, USA) was filled with a vinyl polysiloxane impression material that had a putty consistency to create a silicon mold. The reference denture was then placed into the impression material, keeping the denture teeth parallel to the bottom of the flask. This caused the impression material to extrude flush with the denture’s periphery. With a scalpel, the extra duplication material was removed, and the impression material’s border was perforated at two locations to resemble the wax sprues. Then, the separating fluid was added to the denture and the adjacent impression material. The reference CD’s intaglio side and the upper half of the duplicating flask were both filled with putty-like vinyl polysiloxane impression material. Then the flask was closed, and hand-tightening of the closure screw produced an impression mold. After the impression materials were completely set, the denture and surrounding wax sprues were removed. Inside a furnace (TCS Dental, Inc., Signal Hill, CA, USA), the thermoplastic resin was heated to between 270 and 280 ℃ for 18 min and then automatically injected into one of the main apertures at 8.5 bar of pressure until the other opening overflowed. The flasks were slowly cooled, and the duplicated dentures were removed. 

The resulting 50 mandibular CDBs created using different manufacturing methods were finished and polished according to each manufacturer’s instructions. 

Accuracy measurement: The reference denture (Figure 1) was scanned using a laboratory scanner. Each duplicated denture was fixed to the scanning table by affixing two pieces of plasticine to the lower borders of the dentures. The same laboratory scanner was used for the duplicated dentures. The distances between reference points on the fitting surface’s STL file of the reference model and the STL files of the 50 CDs were measured using Mimics software (Mimics Innovation Suite, version 13.1, Materialise HQ, Leuven, Belgium), enabling comparisons between them.

The linear measurements (Figure 2) were evaluated separately by two assessors. Each measurement was taken from the center of one reference mark to the center of the other. The second assessor repeated the first assessor’s measurement evaluation in the same manner. The mean of the two readings for each measurement was recorded, and an intra-class correlation coefficient test was conducted to find the level of interobserver dependability.

**Figure 3:**
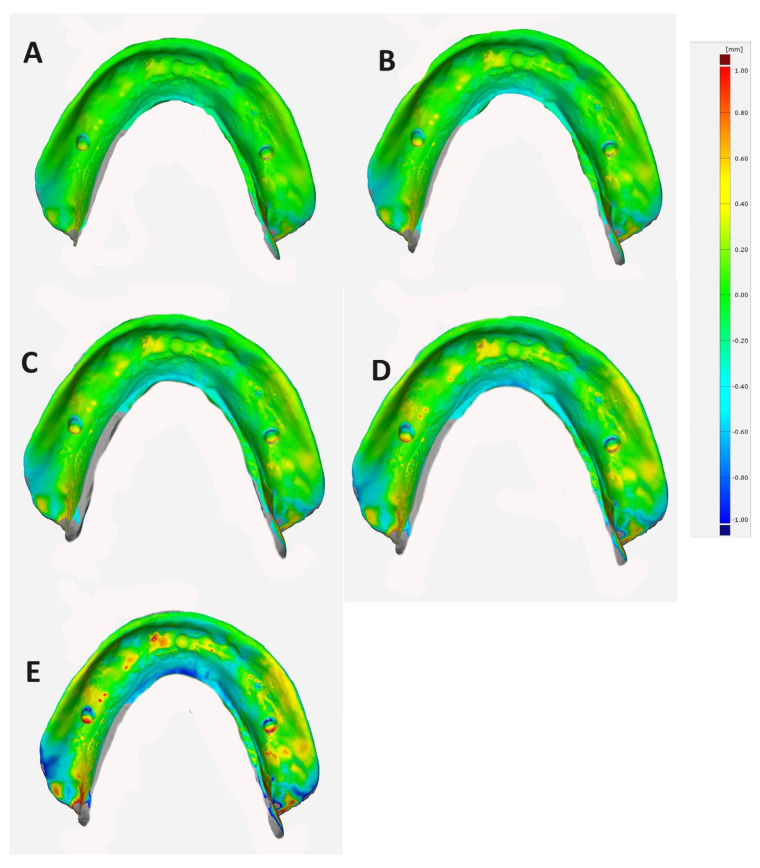
Representative images showing color maps and deviations of different mandibular denture bases using superimposition evaluation method. (**A**) AvaDent; (**B**) IvoBase; (**C**) NextDent; (**D**) Harz Lab; and (**E**) injected CDB. Yellow to red indicates impingement of the CDB on the model, blue indicates space between the duplicated CD and reference model, and green indicates contact between the duplicated CD and reference model.

Superimposition technique: The adaptation and correspondence of duplicate CDs with the reference model were evaluated using the superimposition method. The STL file of the reference model underwent inversion. Then, the intaglio surface scans were superimposed using surface matching software (Geomagic Control-X; 3-D-Systems Canada) that created the best-fit alignment. The vertical distances between these superimpositions were subsequently calculated. The outcomes, presented as color maps in Figure 3, indicate the accuracy and adaptability of the fabrication technique, with values closer to 0 signifying higher precision.

Statistical analysis: The data were statistically analyzed using the SPSS ^®^ statistics software version 20. A Shapiro–Wilk test was used to check the normality of the distribution. The data were analyzed using the F-test (one-way ANOVA), and Tukey’s post hoc HSD tests, with the significance level set at *p* ≤ 0.05.

## 3. Results

The Shapiro–Wilk test confirmed the data were normally distributed (*p* > 0.05) for all the studied variables. The one-way ANOVA revealed a significant (*p* < 0.05) effect of the duplication techniques on the accuracy of the fitting surface of the tested CDBs in the A–B and A–C directions (anteroposterior).

The anteroposterior measurements of the reference denture in the A–B and A–C directions were 33.07 mm and 32.00 mm, respectively. In the tested CDBs, the measurements were 33.06 ± 0.11 mm, 32.88 ± 0.08 mm, 32.70 ± 0.07 mm, 32.68 ± 0.08 mm, and 32.51 ± 0.15 mm in the A–B direction, and 31.99 ± 0.07 mm, 31.69 ± 0.09 mm, 31.30 ± 0.07 mm, 31.30 ± 0.12 mm, and 29.38 ± 0.22 mm in the A–C direction for G1, G2, G3, G4, and G5 respectively. The pairwise comparisons using Tukey’s test showed significant differences between the groups in both directions except for between G3 and G4 in the A–B direction (*p* ˃ 0.05).

The one-way ANOVA results demonstrated a statistically significant (*p* < 0.05) impact of the duplication technique on the fitting surface accuracy of tested CDBs in the B–C direction (mediolateral). The mediolateral measurement of the reference denture in the B–C direction was 47.20 mm. For the tested CDBs, the measurements were 47.09 ± 0.14 mm, 46.8 ± 0.02 mm, 46.29 ± 0.04 mm, 45.89 ± 0.07 mm and 45.34 ± 0.15 mm for G1, G2, G3, G4, and G5, respectively. The pairwise comparisons using Tukey’s test showed significant differences between the groups in the B–C measurements (*p* < 0.05).

For all the different linear measurements (A–B, A–C, and B–C directions), the milled CDB groups exhibited significantly fewer dimensional changes than the 3D-printed (G3 and G4) and injection-molded (G5) groups (*p* < 0.05). The injection-molded CDBs showed the most significant dimensional changes (*p* < 0.05; Table 1).

The one-way ANOVA indicated that the duplication method used had a significant influence (*p* < 0.05) on the adaptation of the CDBs. Additionally, Tukey’s pairwise post hoc test highlighted significant differences (*p* < 0.05) between the groups. Among the specimens, the milled CDBs exhibited the least significant deviation in mean values (0.155 ± 0.004 mm and 0.191 ± 0.002 mm for Avadent and Ivobase, respectively) and demonstrated the closest adaptation to the reference model (*p* < 0.05). The 3D-printed specimens displayed higher mean deviation values (0.236 ± 0.111 mm and 0.296 ± 0.005 mm for NextDent and HarzLab, respectively) than the milled specimens. However, the injection-molded (iFlext) specimens demonstrated the most significant deviation in mean values (0.626 ± 0.186 mm), indicating they had the lowest adaptation to the reference model among all the specimens (Table 2).

## 4. Discussion

Denture replication is an excellent method for conventional denture replacement as it requires minimum fabrication time and involves fewer patient complaints as neuromuscular adaptation is easier than in a new CCD [7]. Clinicians can recreate a medically effective CD using conventional or digital methods [1,25,26]. However, the traditional approach requires significant effort and time [1]. Therefore, in the present study, an advanced CAD-CAM processing technique was selected to examine the accuracy of CAD-CAM milling versus 3D printing in denture duplication. In the conventional duplicating technique, a mold that serves as the negative representation of the CD is made using irreversible hydrocolloid or elastomeric impression materials. The entire duplication process must be finished quickly to prevent distortion [1]. To prevent the bias of mold deformation, elastomeric impression material was used in this study rather than hydrocolloid impression material. 

Traditionally, an impression is taken or a flasking-and-investing procedure is used to duplicate a denture, after which the resin is repositioned using heat- or self-curing [27]. In this study, the mandibular master CD reference was selected due to its limited undercuts and ease of scanning using an optical scanner (laboratory scanner). CDs featuring substantial undercuts and extensive denture extensions might pose challenges regarding their optical scanning feasibility [1,25,28,29]. The current data demonstrated the presence of significant differences in the accuracy of the fitting surfaces between the tested CDBs (*p* ˂ 0.001). Therefore, the null hypothesis was rejected. 

Chen et al. [1] reported that the trimming, finishing, and total labor time spent on the digital light processing (DLP) 3D printer groups in their study was significantly less than the stereolithography (SLA) 3D printer groups. Therefore, the DLP 3D printer was chosen in the current study instead of the SLA 3D printer for printing the duplicated dentures because it is fast and more accurate [30]. The printed denture bases were rinsed in 99% isopropyl ethyl alcohol for 5 min to remove any uncured resin material; however, increased rinsing time could result in printed object fissuring [31,32]. The printed dentures were dried and placed in a UV light-curing unit for 30 min to obtain full polymer conversion [31,32]. This technique’s viability has been assessed in prior research [25,33]. In the current study, a 5-axis CAD-CAM milling method was selected for denture duplication using pre-polymerized blocks of PMMA and computer software, as the 5-axis machines are appropriate for generating complex structures, such as partial denture frameworks, acrylic denture bases, and screw-retained implant prostheses in subtractive manufacturing [34]. 

In the current study, linear distortion was chosen to represent the accuracy of the duplicated dentures produced by the different processing techniques as during processing, dentures can have linear deformations of 0.45–0.9%. Such deformations can result in the reduced adaptation of the denture base to the mucosa [6]. However, linear measurements between points do not account for how manufacturing deformation affects the intricate 3D structure of a denture base. Therefore, the method’s clinical applicability is debatable [35]. In addition to the linear measuring method, the best-fit alignment method (superimposition) was used to measure the deviation of the duplicated denture bases, as this method enables the overlaying and analysis of scanned files using advanced software. Previous studies have demonstrated that these evaluation techniques are valid [1,6,35]. In contrast to linear or cross-sectional measurements (2D), which provide limited information, the 3D AutoCAD software technique produces a more accurate picture of the overall deformation across the complete fitting surface of the denture base. This is why it was selected as a test technique in the present study [35]. 

The current findings revealed that the dimensional accuracy of the CAD-CAM milled group was statistically greater than the other two 3D-printed groups. This outcome is supported by the results of previous investigations [10,36]. This mismatch can be attributed to the internal tensions created after polymerization and shrinkage during polymerization. While industrially pre-polymerized PMMA pucks of the final dimensions are subtracted during the milling process, photopolymerized 3D printing is influenced by polymerization shrinkage [36]. The statistically significantly different linear measures between the 3D-printed resin and CAD-CAM-milled dentures reported in the current study could be due to the chosen build angle (90°) of the resin pattern and thick support, which may have impacted the resin pattern’s dimensional accuracy and, ultimately, the overall accuracy, as reported by Negm et al. [23], and Alharbi et al. [37]. 

This investigation revealed significant differences in the linear measurements between CAD-CAM milled dentures and 3D-printed resin dentures. The use of a high-precision 5-axis CAD-CAM milling machine compared to a DLP 3D printer with a 50 µm layer and three axes might explain these disparities. Consequently, CAD-CAM-milled dentures exhibited superior dimensional accuracy over 3D-printed dentures [36,38]. Furthermore, the polymerization process involved in creating 3D-printed dentures, which occurs during the final light-polymerization step, could potentially result in polymerization shrinkage. This phase might cause distortion during the removal of partially polymerized dentures from the build platform or during the isopropyl alcohol wash and polymerization procedure. However, the complexities of the printing process and uncertainties surrounding duplicated fault recovery make it difficult to pinpoint the specific processing issues [36].

The injected method is a conventional method used for denture fabrication and demonstrated the lowest accuracy between the tested groups. This may be attributed to the drawbacks associated with conventional methods compared to digital methods that have been reported in the literature. It is likely this low accuracy primarily resulted from polymerization shrinkage and associated internal polymerization stress [10,39]. This is in agreement with the findings of Li et al. [40] and Lee et al. [10], who compared the accuracy of the milled, 3D-printed, and injected methods, and reported that the milled technique demonstrated the highest accuracy, followed by 3D printed technique, with the injected method showing the lowest accuracy. Similarly, Einarsdottir et al. [41] compared the accuracy of CAD-CAM milled, injected, and compression-molding methods, and they reported that the CAD-CAM milled method showed significantly fewer dimensional changes compared with the other methods, which is in line with the findings of the current study.

Improved cost-effectiveness, decreased visits, decreased chair time, and the availability of recorded data for any required adjustments or future procedures are some of the benefits of digital duplication techniques. The CAD-CAM milling and 3D-printed technologies for denture duplication demonstrated fewer dimensional changes and higher accuracy compared with conventional denture duplication techniques. The deviations noted for all the tested denture base materials using different duplication techniques were within the clinically acceptable value, being less than 1 mm [36]. Regarding the need to implement a digital method for removable prostheses fabrication, the CAD-CAM milled or 3D printed duplicated dentures are clinically suitable solutions for denture duplication and valid alternatives to the injection-molding technique. CAD-CAM monolithic CDs present a better denture base for teeth bonding than 3D-printed ones [42]. Therefore, one-piece milled CDs and conventional CDs seem to be a safer choice [43].

The absence of oral condition simulations, thermocycling, and long-term water storage are considered the main limitations of the current study. Future studies should investigate the effect of wet conditions, long-term water storage, and thermocycling on the fitting surface accuracy and mechanical and physical properties of CAD-CAM milled, 3D-printed, and injection molded CDBs.

## 5. Conclusions

Duplicating CDs using the CAD-CAM milling technique produced superior fitting surface accuracy than using 3D-printed and injected-molded techniques. However, in a therapeutic situation, this difference might not be noticeable. The CAD-CAM milling duplication technique could prove useful for producing duplicate CDs with a high degree of accuracy. Despite their positive results, the digital duplication procedures differed significantly between dentures.

## Figures and Tables

**Table 1:** One-way ANOVA and pairwise comparisons tests between different groups in concern linear measurement (mm) method.

Measurement Points		ANOVA Test				Tukey’s Post Hoc Test	
		Variable		Mean + SD (mm)	*p*	Pairwise-Comparison	*p*
Antero-posterior	A–B	Reference Denture	1	(33.07)	0.000 *	G 1 vs. G 2	0.012
		G1 (AvaDent)	10	33.06 ± 0.11		G 1 vs. G 3	0.000
		G2 (IvoBase)	10	32.88 ± 0.08		G 1 vs. G 4	0.000
		G3 (NextDent)	10	32.70 ± 0.07		G 1 vs. G 5	0.000
		G4 (Harzlab)	10	32.68 ± 0.08		G 2 vs. G 3	0.013
		G5 (iFlext)	10	32.51 ± 0.15		G 2 vs. G 4	0.006
						G 2 vs. G 5	0.000
						G 3 vs. G 4	0.764
						G 3 vs. G 5	0.010
						G 4 vs. G 5	0.019
	A–C	Reference Denture	1	(32.00)	0.000 *	G 1 vs. G 2	0.002
		G 1	10	31.99 ± 0.07		G 1 vs. G 3	0.000
		G 2	10	31.69 ± 0.09		G 1 vs. G 4	0.000
		G 3	10	31.30 ± 0.07		G 1 vs. G 5	0.000
		G 4	10	31.30 ± 0.12		G 2 vs. G 3	0.000
		G 5	10	29.38 ± 0.22		G 2 vs. G 4	0.000
						G 2 vs. G 5	0.000
						G 3 vs. G 4	0.000
						G 3 vs. G 5	0.000
						G 4 vs. G 5	0.000
Medio-lateral	B–C	Reference Denture	1	(47.20)	0.000 *	G 1 vs. G 2	0.000
		G 1	10	47.09 ± 0.14		G 1 vs. G 3	0.000
		G 2	10	46.8 ± 0.02		G 1 vs. G 4	0.000
		G 3	10	46.29 ± 0.04		G 1 vs. G 5	0.000
		G 4	10	45.89 ± 0.07		G 2 vs. G 3	0.000
		G 5	10	45.34 ± 0.15		G 2 vs. G 4	0.000
						G 2 vs. G 5	0.000
						G 3 vs. G 4	0.000

* Significant at the 0.05 level.

**Table 2:** One-way-ANOVA and pairwise associations tests between different groups concerning dimensional changes in the superimposition method.

Denture Base Resins		Mean ± SD Mm	ANOVA Test	Tukey’s Post Hoc Test	
Milled	G 1	0.155 ± 0.004	0.000	G 2	0.000
				G 3	0.000
				G 4	0.000
				G 5	0.000
	G 2	0.191 ± 0.002		G 1	0.000
				G 3	0.000
				G 4	0.000
				G 5	0.000
3D Printed	G 3	0.236 ± 0.111		G 1	0.000
				G 2	0.000
				G 4	0.000
				G 5	0.000
	G 4	0.296 ± 0.005		G 1	0.000
				G 2	0.000
				G 3	0.000
				G 5	0.000
Injected	G 5	0.626 ± 0.186		G 1	0.000
				G 2	0.000
				G 3	0.000
				G 4	0.000

## Data Availability

The datasets that support the findings of this study are available from the corresponding author upon reasonable request.
